# Testing Differential Gene Networks under Nonparanormal Graphical Models with False Discovery Rate Control

**DOI:** 10.3390/genes11020167

**Published:** 2020-02-05

**Authors:** Qingyang Zhang

**Affiliations:** Department of Mathematical Sciences, University of Arkansas, Arkansas, AR 72701, USA; qz008@uark.edu

**Keywords:** gene regulatory network, nonparanormal graphical model, network substructure, false discovery rate control

## Abstract

The nonparanormal graphical model has emerged as an important tool for modeling dependency structure between variables because it is flexible to non-Gaussian data while maintaining the good interpretability and computational convenience of Gaussian graphical models. In this paper, we consider the problem of detecting differential substructure between two nonparanormal graphical models with false discovery rate control. We construct a new statistic based on a truncated estimator of the unknown transformation functions, together with a bias-corrected sample covariance. Furthermore, we show that the new test statistic converges to the same distribution as its oracle counterpart does. Both synthetic data and real cancer genomic data are used to illustrate the promise of the new method. Our proposed testing framework is simple and scalable, facilitating its applications to large-scale data. The computational pipeline has been implemented in the R package *DNetFinder*, which is freely available through the Comprehensive R Archive Network.

## 1. Background

Inferring the structural change of a network under different conditions is essential in many problems arising in biology, medicine, and other scientific fields. For instance, in genomics, it is often of importance to study the structural change of a genetic pathway between diseased and normal groups. In the field of brain mapping, it is critical to identify the difference in brain connectivity between groups (for example, the brain connectivity network of normal subjects and patients often possess different structures). Most of these applications have relied on the prevailing Gaussian graphical models (GGMs) because of its good interpretability and computational convenience, and there is a rich and growing literature on learning differential networks under GGMs. To name a few, Guo et al. (2015) [1] introduced a joint estimation for multiple GGMs by a group lasso approach, under the assumption that the GGMs being studied are sparse and only differ in a small portion of edges. Danaher et al. (2014) [2] proposed a fused graphical lasso method which is free from the sparsity assumption on condition-specific networks and only requires the sparsity of the differential network. Zhao et al. (2014) [3] constructed a new estimator which directly estimates the differential network defined as $\Delta =\Sigma_{X}^{-1}-\Sigma_{Y}^{-1}$, where $\Sigma_{X}^{-1}$ and $\Sigma_{Y}^{-1}$ represent the two condition-specific precision matrices and Δ, $\Sigma_{X}^{-1}$, $\Sigma_{Y}^{-1}$ have the same dimension. Liu (2017) [4] presented a new test to simultaneously study structural similarities and differences between multiple high-dimensional GGMs, which adopts the partial correlation coefficients to characterize the potential changes of dependency strength between two variables.

Most of the aforementioned algorithms were based upon penalized likelihood maximization. Although some algorithms were consistent under certain regularity conditions, they failed to control the false discovery rate (FDR) of the substructure detection as it is difficult to choose a tuning parameter to control the FDR at the desired level [1,2,3]. One exception is Liu (2017), who introduced a hierarchical testing framework to adjust for the multiplicity. Liu’s test was constructed to asymptotically control the FDR while keeping satisfactory statistical power. Simulation studies in [4] have shown that this new test exhibits substantial power gains over existing methods such as graphical lasso. One major drawback that limits the application of Liu’s test is the Gaussian assumption, which is often violated in practice especially in genomics. For instance, some digital measurements of gene expression level such as RNA-Seq data often greatly deviate from normality even after log-transformation or other variance-stabilizing transformations. In this paper, we aim to extend Liu’s work to a more flexible semiparametric framework, namely the nonparanormal graphical models (NPNGMs), where the random variables are assumed to follow a multivariate normal distribution after a set of monotonically increasing transformations. We use a novel rank-based multiple testing method to detect the structural difference between multiple networks from non-Gaussian data. The method is computationally efficient and asymptotically controls the FDR at a desired level. To begin with, we give the formal definition of nonparanormal distribution:

**Definition** **1.** *A random vector $Y=(Y_{1},Y_{2},...,Y_{p})$ follows a nonparanormal distribution if there exists a set of univariate and monotonically increasing transformations, $f=(f_{1},...,f_{p})$, such that:*

$$\left(X_{1},...,X_{p}\right)\equiv \left(f_{1}\left(Y_{1}\right),...,f_{p}\left(Y_{p}\right)\right)∼N\left(\mu ,\Sigma \right),$$

*where **μ** and* Σ *denote the mean and covariance matrix in the multivariate normal distribution, respectively. The distribution of Y depends on three parameters and it can be generally written as Y∼NPN(μ,Σ,f).*

By Definition 1 and Sklar’s theorem, it is easy to verify that when the transformation functions $f_{j}^{\prime }s$ are all differentiable, the nonparanormal distribution NPN(μ,Σ,f) is equivalent to a Gaussian copula [5]. As graphical models, the NPNGMs are much more flexible than GGMs in modeling non- Gaussian data while retaining the interpretability of the latter. Some recent studies have established the estimation and properties of high dimensional nonparanormal graphical models. For example, Liu et al. (2009) [5], who first studied high-dimensional NPNGMs, bridged the estimations of GGMs and NPNGMs by a nonparametric and truncated (Winsorized) estimator of the unknown transformation functions. Xue and Zou (2012) [6] proposed to use an adjusted Spearman’s correlation to estimate the structure of high-dimensional NPNGMs, and they showed that the rank-based estimator achieves the same rate of convergence as its oracle counterpart (i.e., assuming known transformation functions). Despite the advances in single NPNGM estimation, to the best of our knowledge, the inference of differential substructure between multiple NPNGMs has not been studied. In this paper, we tackled this problem by embedding the Winsorized estimator into the testing framework of Liu (2017). Under some regularity conditions, we showed that the new test statistic converges to the same distribution as its oracle counterpart does [4].

We begin with the notations and problem formulation. For a vector $a=(a_{1},...,a_{p})$, we define its $\ell_{0}$ norm as ${∥a∥}_{\ell_{0}}=\sum_{i=1}^{p}I\left\{a_{i}\neq 0\right\}$, its $\ell_{1}$ norm as ${∥a∥}_{\ell_{1}}=\sum_{i=1}^{p}\left|a_{i}\right|$, its $\ell_{2}$ norm as ${∥a∥}_{\ell_{2}}=\sqrt{\sum_{i=1}^{p}a_{i}^{2}}$, and its $\ell_{\infty }$ norm as ${∥a∥}_{\ell_{\infty }}=\max_{i}\left|a_{i}\right|$. For a matrix $A=\left(a_{ij}\right)\in R^{p\times q}$, we define its $\ell_{0}$ norm as ${∥A∥}_{0}=\sum_{i,j}I\left\{a_{ij}\neq 0\right\}$, its $\ell_{1}$ norm as ${∥A∥}_{1}=\sum_{i,j}\left|a_{ij}\right|$, its Frobenius norm as ${∥A∥}_{F}=\sqrt{\sum_{i,j}a_{ij}^{2}}$ and its $\ell_{\infty }$ norm as ${∥A∥}_{\infty }=\max_{i,j}\left|a_{ij}\right|$. Let $A_{i,-j}$ denote the *i*th row of A with its *j*th entry being removed and $A_{-i,j}$ denote the *j*th column with its *i*th entry being removed. We use $A_{-i,-j}$ to denote a (p−1)×(q−1) matrix by removing the *i*th row and the *j*th column. For square matrix B, we let $\lambda_{\max}\left(B\right)$ and $\lambda_{\min}\left(B\right)$ denote the largest and smallest eigenvalues of B respectively. In addition, for a given sequence of random variable $\left\{X_{n},n=1,2,...\right\}$ and a constant sequence $\left\{a_{n},n=1,2,...\right\}$, $X_{n}=o_{p}\left(a_{n}\right)$ denotes that $X_{n}/a_{n}$ converges to zero in probability as *n* approaches to infinity and $X_{n}=O_{p}\left(a_{n}\right)$ denotes that $X_{n}/a_{n}$ is stochastically bounded. If there are positive constants *c* and *C* such that $c\leq X_{n}/a_{n}\leq C$ for all n≥1, we write $X_{n}∼a_{n}$.

To formulate the problem, we let k∈{1,2,...,K} be the index of class, *p* be the dimension, and $\left(Y_{1}^{\left(k\right)},...,Y_{n_{k}}^{\left(k\right)}\right)$ be a sample of size $n_{k}$ for class *k* where $Y_{m}^{\left(k\right)}={\left(Y_{m1}^{\left(k\right)},...,Y_{mp}^{\left(k\right)}\right)}^{T}\in R^{p}$, $m\in \{1,...,n_{k}\}$. Under $Y_{m}^{\left(k\right)}∼NPN\left(\mu^{\left(k\right)},\Sigma^{\left(k\right)},f^{\left(k\right)}\right)$, we test the following hypothesis:

$$\begin{array}{cc} H_{0ij}:\; & \rho_{ij\cdot }^{\left(1\right)}=\rho_{ij\cdot }^{\left(2\right)}=...=\rho_{ij\cdot }^{\left(K\right)},\\ H_{aij}:\; & \rho_{ij\cdot }^{\left(k\right)}\neq \rho_{ij\cdot }^{\left(k^{\prime }\right)},\;\mathrm{for}\;\mathrm{some}\;k,k^{\prime }\in \left\{1,...,K\right\}, \end{array}$$

where 1≤i,j≤p, ${\left\{\Sigma^{\left(k\right)}\right\}}^{-1}=\Omega^{\left(k\right)}=\left(\omega_{ij}^{\left(k\right)}\right)$, and $\rho_{ij\cdot }^{\left(k\right)}$ represents the partial correlation coefficient between $X_{i}^{\left(k\right)}$ and $X_{j}^{\left(k\right)}$ given $X^{\left(k\right)}\setminus \left(X_{i}^{\left(k\right)},X_{j}^{\left(k\right)}\right)$, $\left(X_{m1}^{\left(k\right)},...,X_{mp}^{\left(k\right)}\right)=\left(f_{1}^{\left(k\right)}\left(Y_{m1}^{\left(k\right)}\right),...,f_{p}^{\left(k\right)}\left(Y_{mp}^{\left(k\right)}\right)\right)$. The edge (i,j) is a differential edge if $\rho_{ij\cdot }^{\left(k\right)}\neq \rho_{ij\cdot }^{\left(k^{\prime }\right)}$ for some $k,k^{\prime }\in \left\{1,...,K\right\}$, and the differential network is defined as the set of all differential edges. As a well-known result in statistics, $\rho_{ij\cdot }^{\left(k\right)}=-\omega_{ij}^{\left(k\right)}/\sqrt{\omega_{ii}^{\left(k\right)}\omega_{jj}^{\left(k\right)}}$. Here, we consider an equivalent alternative of the hypothesis testing above. Similar as in [4], let

(1) $$S_{ij}\left(\Omega \right)=\sqrt{\sum_{1\leq k<k^{\prime }\leq K}{\left(\rho_{ij\cdot }^{\left(k\right)}-\rho_{ij\cdot }^{\left(k^{\prime }\right)}\right)}^{2}},$$

then the hypothesis testing can be simplified as

$$\begin{array}{cc} H_{0ij}:\; & S_{ij}\left(\Omega \right)=0,\\ H_{aij}:\; & S_{ij}\left(\Omega \right)>0. \end{array}$$

As $S_{ij}\left(\Omega \right)=S_{ji}\left(\Omega \right)$, we define $H_{0}=\left\{H_{0ij},1\leq i<j\leq p\right\}$ and $H_{a}=\left\{H_{aij},1\leq i<j\leq p\right\}$, and the total numbers of tests are p(p−1)/2, i.e., $card\left(H_{0}\right)=card\left(H_{a}\right)=p\left(p-1\right)/2$.

The rest of this paper is structured as follows: In Section 2, we introduce the new test statistic and multiple testing procedure. In Section 3 we perform a simulation study to evaluate the finite sample performance of the proposed test in terms of FDR control and statistical power. We then apply the new method to a rich genomic data to study the genetic difference between four breast cancer subtypes. We discuss the strength and shortcomings of the test in Section 5. Technical proof of the asymptotic results is provided in Appendix A.

## 2. Statistical Methods

### 2.1. Winsorized Estimator of the Latent Gaussian Variables

In practice, the transformation functions $f^{\left(k\right)}=\left(f_{1}^{\left(k\right)},...,f_{p}^{\left(k\right)}\right)$ in the nonparanormal distribution are unknown. However, one can use a Winsorized estimator to approximate $f^{\left(k\right)}$, i.e., to impute the latent Gaussian variables (oracle data) ${\left(X_{m1}^{\left(k\right)},...,X_{mp}^{\left(k\right)}\right)}_{1\leq m\leq n_{k}}$. To illustrate the Winsorized estimator, we define the following quantile function:

$${\hat{h}}_{j}^{\left(k\right)}\left(t\right)=\Phi^{-1}\left({\tilde{F}}_{j}^{\left(k\right)}\left(t\right)\right),\;1\leq j\leq p,$$

where ${\tilde{F}}_{j}^{\left(k\right)}$ is some estimator of the cumulative distribution function of $Y_{j}^{\left(k\right)}$, and a natural choice for ${\tilde{F}}_{j}^{\left(k\right)}$ would be the empirical cumulative distribution function (eCDF)

$${\hat{F}}_{j}^{\left(k\right)}\left(t\right)=\frac{1}{n_{k}}\sum_{m=1}^{n_{k}}I\left\{Y_{mj}^{\left(k\right)}\leq t\right\}.$$

One major drawback of the eCDF above is that under high dimensionality, the variance of ${\hat{F}}_{j}^{\left(k\right)}\left(t\right)$ could be too large. To overcome the problem, Liu et al. (2009) considered a truncated (Winsorized) estimator as follows:

$${\tilde{F}}_{j}^{\left(k\right)}=\left\{\begin{array}{cc} \delta_{n} & {\hat{F}}_{j}^{\left(k\right)}\left(t\right)<\delta_{n}\\ {\hat{F}}_{j}^{\left(k\right)}\left(t\right) & \delta_{n}\leq {\hat{F}}_{j}^{\left(k\right)}\left(t\right)\leq 1-\delta_{n},\\ 1-\delta_{n} & {\hat{F}}_{j}^{\left(k\right)}\left(t\right)>1-\delta_{n} \end{array}\right.$$

where $\delta_{n}$ serves as the truncation parameter that should be carefully chosen. Liu et al. (2009) [5] suggested $\delta_{n}=1/\left(4n^{1/4}\sqrt{\pi \log n}\right)$ to balance the bias and variance of eCDF, and so we will use this value in our calculations. To estimate the transformation functions and impute the latent Gaussian variable X, we define

$$X_{mj}^{(k)*}={\tilde{f}}_{j}^{\left(k\right)}\left(Y_{mj}^{\left(k\right)}\right)={\hat{\mu }}_{j}^{\left(k\right)}+{\hat{\sigma }}_{j}^{\left(k\right)}{\tilde{h}}_{j}^{\left(k\right)}\left(Y_{mj}^{\left(k\right)}\right),$$

where ${\tilde{h}}_{j}^{\left(k\right)}\left(t\right)$, ${\hat{\mu }}_{j}^{\left(k\right)}$ and ${\hat{\sigma }}_{j}^{\left(k\right)}$ are given below:

$${\tilde{h}}_{j}^{\left(k\right)}\left(t\right)=\Phi^{-1}\left({\tilde{F}}_{j}^{\left(k\right)}\left(t\right)\right),$$

$${\hat{\mu }}_{j}^{\left(k\right)}=\frac{1}{n_{k}}\sum_{m=1}^{n_{k}}Y_{mj}^{\left(k\right)},$$

$${\hat{\sigma }}_{j}^{\left(k\right)}=\sqrt{\frac{1}{n_{k}}\sum_{m=1}^{n_{k}}{\left(Y_{mj}^{\left(k\right)}-{\hat{\mu }}_{j}^{\left(k\right)}\right)}^{2}}.$$

The Winsorized estimator $X_{mj}^{(k)*}$ generally works well in approximating the unknown $X_{mj}^{\left(k\right)}$, and it could be used to estimate the oracle sample covariance. Let ${\hat{\Sigma }}^{\left(k\right)}$ be the sample covariance matrix by the oracle data, and ${\tilde{\Sigma }}^{\left(k\right)}$ be the sample covariance matrix by $\left(X_{1}^{(k)*},...,X_{p}^{(k)*}\right)$, that is

$${\tilde{\Sigma }}^{\left(k\right)}=\frac{1}{n_{k}}\sum_{m=1}^{n_{k}}\left(X_{m}^{(k)*}-{\tilde{\mu }}^{\left(k\right)}\right){\left(X_{m}^{(k)*}-{\tilde{\mu }}^{\left(k\right)}\right)}^{T},$$

where ${\tilde{\mu }}^{\left(k\right)}=\left(1/n_{k}\right)\sum_{m=1}^{n_{k}}X_{m}^{(k)*}$. Liu et al. (2009) established the following consistency results under mild regularity conditions:

$${∥{\tilde{\Sigma }}^{\left(k\right)}-{\hat{\Sigma }}^{\left(k\right)}∥}_{\infty }=O_{p}\left(\sqrt{\frac{\log p\log^{2}n_{k}}{n_{k}^{1/2}}}\right).$$

When estimating the precision matrix $\Omega^{\left(k\right)}$, one can consider a modified graphical lasso based on imputed data, i.e.,

(2) $${\tilde{\Omega }}_{glasso}^{\left(k\right)}=\arg\min_{\Omega }\left\{tr\left(\Omega {\tilde{\Sigma }}^{\left(k\right)}\right)-\log|\Omega |+{\lambda ∥\Omega ∥}_{1}\right\}.$$

Liu et al. (2009) showed the following convergence, which elucidated the asymptotic equivalence between the oracle data and imputed data in the structural estimation of NPNGM

$${∥{\tilde{\Omega }}_{glasso}^{\left(k\right)}-\Omega^{\left(k\right)}∥}_{F}=O_{p}\left(\sqrt{\frac{(∥\Omega^{\left(k\right)}{∥}_{0}+p)\log p\log^{2}n_{k}}{n_{k}^{1/2}}}\right).$$

### 2.2. Asymptotic Results for a Single Class

To extend Liu’s test to a nonparanormal case, we first consider the problem of single GGM estimation based on oracle data, i.e., ${\left(X_{m1}^{\left(k\right)},...,X_{mp}^{\left(k\right)}\right)}_{1\leq m\leq n_{k}}∼N\left(\mu_{k},\Sigma_{k}\right)$, in the following regression framework

(3) $$X_{mj}^{\left(k\right)}=\alpha_{j}^{\left(k\right)}+X_{m,-j}^{{\left(k\right)}^{\prime }}\beta_{j}^{\left(k\right)}+\epsilon_{mj}^{\left(k\right)}.$$

It is not hard to show that the regression coefficients $\beta_{j}^{\left(k\right)}=\left(\beta_{j,1}^{\left(k\right)},...,\beta_{j,j-1}^{\left(k\right)},\beta_{j,j+1}^{\left(k\right)},\beta_{j,p}^{\left(k\right)}\right)$ and the error term $\epsilon_{mj}^{\left(k\right)}$ satisfy

$$\beta_{j}^{\left(k\right)}=-{\left(\omega_{jj}^{\left(k\right)}\right)}^{-1}\Omega_{-j,j}^{\left(k\right)},\;\mathrm{cov}\left(\epsilon_{mi}^{\left(k\right)},\epsilon_{mj}^{\left(k\right)}\right)=\frac{\omega_{ij}^{\left(k\right)}}{\omega_{ii}^{\left(k\right)}\omega_{jj}^{\left(k\right)}}.$$

As the oracle data ${\left(X_{m1}^{\left(k\right)},...,X_{mp}^{\left(k\right)}\right)}_{1\leq m\leq n_{k}}$ in Equation (3) are generally unknown, we consider a new regression model based on Winsorized imputations:

(4) $$X_{mj}^{(k)*}={\hat{\alpha }}_{j}^{\left(k\right)}+X_{m,-j}^{\left(k\right){*}^{\prime }}{\hat{\beta }}_{j}^{\left(k\right)}+\epsilon_{mj}^{(k)*}.$$

In solving the problem of single GGM estimation, Liu (2017) proposed an elegant test based on a bias-corrected sample covariance. This has motivated us to construct the following new statistic

(5) $$S_{ij}^{(k)*}=\sqrt{\frac{1}{n_{k}r_{ii}^{(k)*}r_{jj}^{(k)*}}}\left(\sum_{m=1}^{n_{k}}\epsilon_{mi}^{(k)*}\epsilon_{mj}^{(k)*}+\sum_{m=1}^{n_{k}}{\left\{\epsilon_{mi}^{(k)*}\right\}}^{2}{\hat{\beta }}_{i,j}^{\left(k\right)}+\sum_{m=1}^{n_{k}}{\left\{\epsilon_{mj}^{(k)*}\right\}}^{2}{\hat{\beta }}_{j,i}^{\left(k\right)}\right),$$

where $r_{ij}^{(k)*}=\left(1/n_{k}\right)\sum_{m=1}^{n_{k}}\epsilon_{mi}^{(k)*}\epsilon_{mj}^{(k)*}$. By letting ${\bar{\epsilon }}^{\left(k\right)}=\left(1/n_{k}\right)\sum_{m=1}^{n_{k}}\epsilon_{m}^{\left(k\right)}$, ${\left({\hat{\sigma }}_{ij,\epsilon }^{\left(k\right)}\right)}_{1\leq i,j\leq p}=\left(1/n_{k}\right)$$\sum_{m=1}^{n_{k}}\left(\epsilon_{m}^{\left(k\right)}-{\bar{\epsilon }}^{\left(k\right)}\right){\left(\epsilon_{m}^{\left(k\right)}-{\bar{\epsilon }}^{\left(k\right)}\right)}^{\prime }$, $b_{ij}^{\left(k\right)}=\omega_{ii}^{\left(k\right)}{\hat{\sigma }}_{ii,\epsilon }^{\left(k\right)}+\omega_{jj}^{\left(k\right)}{\hat{\sigma }}_{jj,\epsilon }^{\left(k\right)}-1$, we will prove that, under mild conditions (see a detailed proof in Appendix A)

(6) $$S_{ij}^{(k)*}+b_{ij}^{\left(k\right)}\frac{\omega_{ij}^{\left(k\right)}}{\omega_{ii}^{\left(k\right)}\omega_{jj}^{\left(k\right)}}\overset{D}{\to }N\left(0,1+\frac{{\left\{\omega_{ij}^{\left(k\right)}\right\}}^{2}}{\omega_{ii}^{\left(k\right)}\omega_{jj}^{\left(k\right)}}\right).$$

Similar as in [4], the estimated coefficients ${\hat{\beta }}_{j}^{\left(k\right)}$ must satisfy the following conditions:

$${∥{\hat{\beta }}_{j}^{\left(k\right)}-\beta_{j}^{\left(k\right)}∥}_{\ell_{1}}=O_{p}\left(a_{n}^{\left(k\right)}\right),$$

$$\min\left\{\lambda_{\max}^{1/2}\left(\Sigma^{\left(k\right)}\right){∥{\hat{\beta }}_{j}^{\left(k\right)}-\beta_{j}^{\left(k\right)}∥}_{\ell_{2}},\max_{1\leq j\leq p}\sqrt{{\left({\hat{\beta }}_{j}^{\left(k\right)}-\beta_{j}^{\left(k\right)}\right)}^{T}{\hat{\Sigma }}_{-j,-j}^{\left(k\right)}\left({\hat{\beta }}_{j}^{\left(k\right)}-\beta_{j}^{\left(k\right)}\right)}\right\}=O_{p}\left(b_{n}^{\left(k\right)}\right),$$

where

(7) $$a_{n}^{\left(k\right)}=o\left(\sqrt{\log p/n_{k}}\right),\;\;\mathrm{and}\;\;b_{n}^{\left(k\right)}=o\left(n_{k}^{-1/4}\right).$$

Equation (6) is our main result, which is essentially a counterpart of Proposition 3.1 in [4]. The detailed proof is given in Appendix A. The asymptotic result we obtained here suggested that, by an appropriate choice of regression coefficients ${\hat{\beta }}_{j}^{\left(k\right)}$, Liu’s test can be readily extended to a nonparanormal framework by Winsorized imputation. Under GGMs, the condition (7) can be satisfied by several popular shrinkage estimators including lasso estimator and Dantzig selector. For the choice of $\beta_{j}^{\left(k\right)}$ under NPNGMs, one can use the rank-based method introduced by Xue and Zou (2012) [6]. Xue and Zou (2012) showed that the rank-based estimator (e.g., rank-based lasso and rank-based Dantzig selector) achieved exactly the same convergence rate as its oracle counterpart, therefore, it also satisfies our condition (7).

### 2.3. Multiple Testing Procedure for FDR Control

Now we introduce the multiple testing procedure for FDR control based on the single-class result from Equation (6). As suggested in [4], the partial correlation coefficient can be well estimated by a thresholding estimator

$${\hat{\rho }}_{ij.}^{\left(k\right)}=S_{ij}^{\left(k\right)}I\left\{\left|S_{ij}^{\left(k\right)}\right|\geq 2\sqrt{\frac{\log p}{n_{k}}}\right\},$$

and we define the following two-sample test statistics

$$S_{ij}^{\left(k,k^{\prime }\right)}=\frac{S_{ij}^{\left(k\right)}-S_{ij}^{\left(k^{\prime }\right)}}{\sqrt{\frac{1}{n_{k}}{\left(1-{\left\{{\hat{\rho }}_{ij.}^{\left(k\right)}\right\}}^{2}\right)}^{2}+\frac{1}{n_{k^{\prime }}}{\left(1-{\left\{{\hat{\rho }}_{ij.}^{\left(k^{\prime }\right)}\right\}}^{2}\right)}^{2}}}.$$

In the multi-sample case $S_{ij}={\left(S_{ij}^{\left(k,k^{\prime }\right)}\right)}_{1\leq k<k^{\prime }\leq K}$, we consider a sum squared test statistics

$$S_{ij}=\sqrt{\sum_{k<k^{\prime }}{\left\{S_{ij}^{\left(k,k^{\prime }\right)}\right\}}^{2}}.$$

Motivated by [4] (Equations 2.6 and 2.7) and [7], we define the following statistic

$$T_{ij}=\Phi^{-1}P\left(\sqrt{\sum_{i=1}^{M}\lambda_{i}Z_{i}^{2}}\leq S_{ij}\right),$$

and constant $A=\left(P_{0}-{\hat{P}}_{0}\right)/Q_{0}$, where $Z_{i},i=1,...,M$ represent a sequence of *M* i.i.d. standard normal random variables, $P_{0}=2\Phi \left(1\right)-1$, ${\hat{P}}_{0}=2\sum_{1\leq i<j\leq p}I\{|T_{ij}\left|\leq 1\right\}/\left(p^{2}-p\right)$, $Q_{0}=\sqrt{2}\phi \left(1\right)$ and $A\left(t\right)=\left(1+|A\right|\frac{\left|t|\phi (t\right)}{\sqrt{2}\left(1-\Phi \left(t\right)\right)}{)}^{-1}$. For a given $0<\alpha_{0}<1$, let

$$t\left(\alpha_{0}\right)=\inf\left\{t\in R,1-\phi \left(t\right)\leq \frac{\alpha_{0}A\left(t\right)\max\left\{1,\sum_{1\leq i<j\leq p}I\left\{T_{ij}\geq t\right\}\right\}}{(p^{2}-p)/2}\right\}.$$

the FDR can be controlled at level α, if we reject $H_{0ij}:\;S_{ij}\left(\Omega \right)=0$ when $T_{ij}\geq t\left(\alpha_{0}\right)$. One may refer to [7] for the detailed proof about this testing procedure.

Our proposed computational pipeline consisted of three steps: (1) Winsorized imputation for the latent Gaussian variables; (2) rank-based estimation of regression coefficients, and (3) multiple testing with FDR control. On the whole, we put forward a simple procedure to estimate the structural difference between multiple nonparanormal graphical models. The computational pipeline for a two-sample comparison has been implemented in the R package *DNetFinder*, which can be downloaded from the Comprehensive R Archive Network (CRAN).

## 3. Numerical Study

We performed a simulation study to evaluate the finite sample performance of the proposed procedure. In particular, we evaluated the empirical false discovery rate (eFDR) as well as the statistical power under two classes, i.e., K=2. The dimension and sample size were set to be p=200 and $n_{1}=n_{2}=100$. We consider two commonly used graph-generating models including the band graph and Erdos–Rényi (ER) graph, and two estimators for regression coefficients including lasso estimator and Dantzig selector. Detailed set-up for precision matrices $\Omega_{1}$ and $\Omega_{2}$ are given below:

- **Band graph:**$\Omega_{1}={\left(\omega_{ij}\right)}_{1\leq i,j\leq p}$ was obtained by the following assignments
  $$\omega_{ij}=\left\{\begin{array}{cc} 1 & |i-j|=0\\ 0.6 & |i-j|=1\\ 0 & |i-j|\geq 2 \end{array}\right..$$
  We then randomly picked 50 edges in $\Omega_{1}$ as the differential edges and changed their signs in $\Omega_{2}$. To ensure positive definiteness, we added $\max(|\lambda_{\min}\left(\Omega_{1}\right)|,|\lambda_{\min}\left(\Omega_{2}\right)|)+0.05$, to the diagonal of $\Omega_{1}$ and $\Omega_{2}$.
- **Erdos–Rényi (ER) graph:** Each node pair (i,j) were randomly connected with probability 5%. A correlation coefficient is generated for each edge in the network from a two-part uniform distribution [−1/2,−1/4]∪[1/4,1/2]. To ensure positive-definiteness, we shrunk the correlations by a factor of 5 and the diagonals were set to be one for $\Omega_{1}$. We then randomly selected 5% of the edges as the differential edges, and changed their signs in $\Omega_{2}$.

For each graph, we generated the latent Gaussian data (oracle data) from $N(0,\Omega^{-1})$, $\Omega \in \{\Omega_{1},\Omega_{2}\}$, and a Winsorized estimator with truncation parameter $\delta_{n}=1/\left(4n^{1/4}\sqrt{\pi \log n}\right)$ was used to implement our test. The performance of the proposed method was then evaluated in two aspects: false discovery rate control and statistical power. In particular, we compared the results based on oracle data and imputed data by the Winsorized estimator. Two estimators including the lasso estimator and Dantzig selector were used to estimate coefficients $\hat{\beta }$. For oracle data, we applied the R package *flare* to calculate the solution path over a sequence of 20 candidate λ’s and tune by Akaike information criterion (AIC). For imputed data, we adopted the rank-based methods introduced by [6], i.e., the rank-based lasso and rank-based Dantzig selector. The simulation was repeated for 100 times for each FDR level (α∈{0.05,0.10,0.15,...,0.50}) and the average empirical FDR and statistical power were summarized.

**Figure 1:**
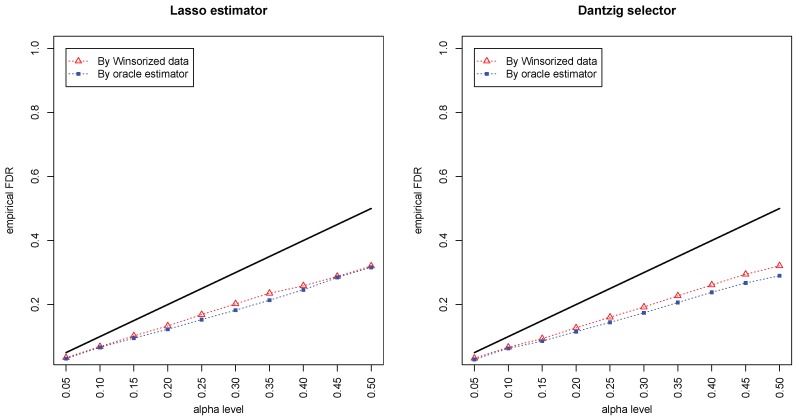
Empirical false discovery rates (eFDRs) by oracle data and Winsorized imputations under the band graph setting. The *x*-axis represents the desired FDR levels from 0.05 to 0.5, and the solid line is y=x.

**Figure 2:**
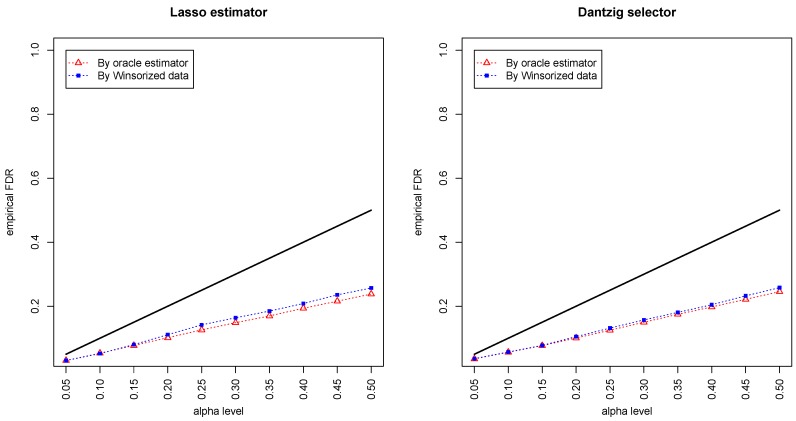
Empirical FDRs (eFDRs) by oracle data and Winsorized imputations under the Erdos–Rényi (ER) graph setting. The *x*-axis represents the desired FDR levels from 0.05 to 0.5, and the solid line is y=x.

Figure 1 and Figure 2 compared the empirical false discovery rate (eFDR) with the desired level α under the band graph and ER graph. It can be seen that the empirical FDR based on imputed data is close to the one by oracle data, both close to the desired level of α, suggesting that the FDRs were controlled quite well for both cases. The lasso estimator works almost equally well as Dantzig selector in both settings.

**Figure 3:**
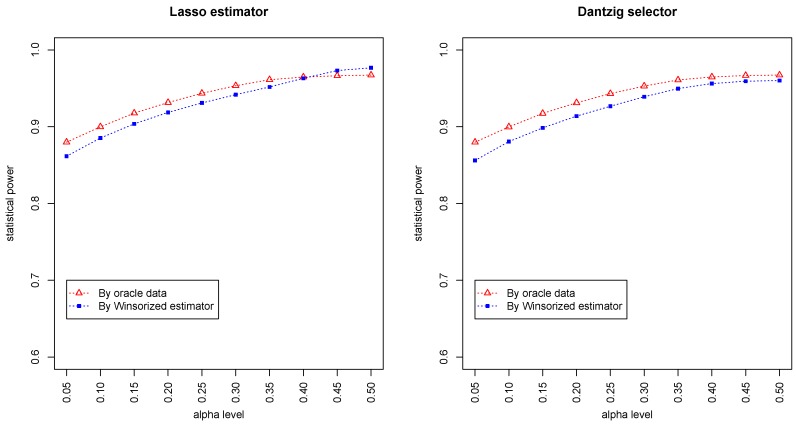
Statistical powers by oracle data and Winsorized imputations under the band graph setting. The *x*-axis represents the desired FDR levels from 0.05 to 0.5.

**Figure 4:**
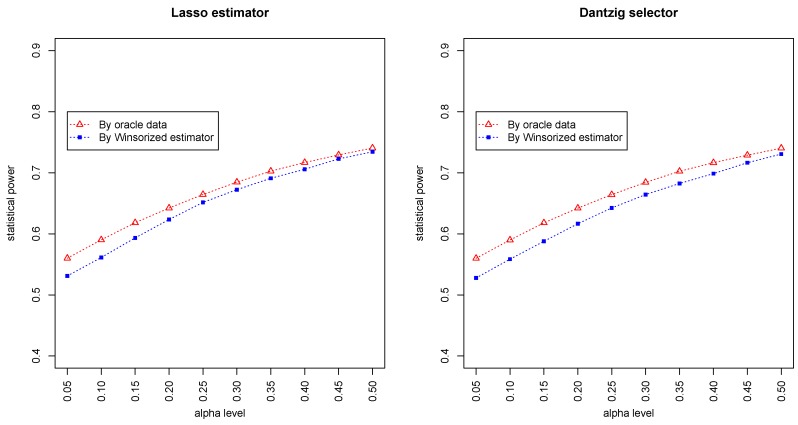
Statistical powers by oracle data and Winsorized estimator under the ER graph setting. The *x*-axis represents the desired FDR levels from 0.05 to 0.5.

Figure 3 and Figure 4 summarized the statistical power of the test for the band graph and ER graph. As can be seen, the power for ER graph is substantially lower than the band graph, indicating that the complexity and denseness of the underlying differential network may significantly decrease the power of our test. The test based on oracle data performs slightly better than the imputed data, which is due to the loss of information during Winsorized imputation. Similar as we observed from Figure 1 and Figure 2, the lasso estimator works almost equally well as Dantzig selector.

**Figure 5:**
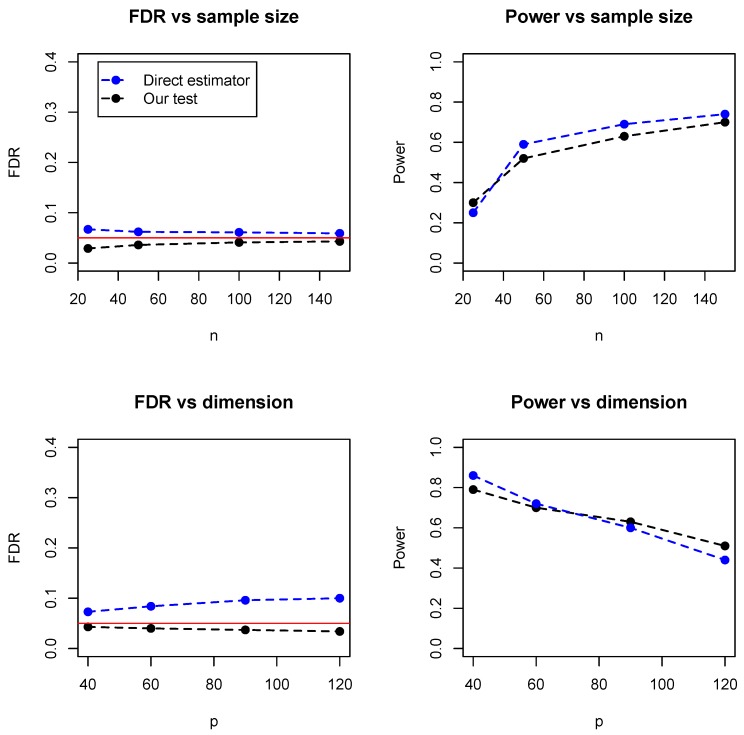
Comparison of the proposed test and direct estimator by Zhang (2019), in terms of empirical FDR and statistical power under different sample sizes and dimensions.

In addition, we compared the proposed test with a direct estimator, recently developed by Zhang (2019) [8]. The direct estimator is a rank-based estimator and can be solved by a parametric simplex algorithm. We simulated the data from the Erdos–Rényi (ER) graph with different sample sizes (n=25,50,100,150) and numbers of dimensions (p=40,60,90,120). As the direct estimator does not control the false discovery rate, we set the FDR level at 0.05 for our proposed test. Figure 5 summarized the empirical FDR and statistical power under different sample sizes (with dimension fixed at 100) and different dimensions (with sample size fixed at 100). It can be seen that the two methods have comparable performance and our proposed test achieves lower FDR but slightly lower statistical power. However, it is noteworthy that the direct estimator is computationally expensive and becomes impractical when the dimensions exceed 150. Table 1 summarized the running time of the two methods, where it can be seen that our test is much faster than the direct estimator, especially for relatively high dimensions. For instance, when p=120, the direct estimator takes hours while our test takes less than 10 seconds. As the core part of the proposed algorithm is the estimation of regression coefficients, the time complexity is the same as the linear regression. For instance, with LASSO and p>n, the time complexity is $O(np^{2})$, while the direct estimator by Zhang (2019) has a time complexity $O(np^{4})$.

## 4. A Genomic Application

In this part, we applied the proposed test to the Cancer Genome Atlas data (TCGA, [9]) to study the different roles of the cell cycle pathway in the two subtypes of breast cancer including luminal A subtype and basal-like subtype. The cell cycle pathway is known to play a critical role in the initiation and progression of many human cancers including breast cancer and ovarian cancer [10,11]. For instance, the cell cycle pathway provided by KEGG (Kyoto Encyclopedia of Genes and Genomes, [12]) contains 128 important genes that co-regulate cell proliferation, including *ATM*, *RB1*, *CCNE1*, and *MYC*. Abnormal regulation among these genes may cause the over-proliferation of cells and an accumulation of tumor cell numbers [11].

The transcriptome profiling data for breast cancer were downloaded through the Genomic Data Commons portal [13] in January 2017. The expression level of each gene was quantified by the count of reads mapped to the gene. The quantifications were done by software *HTSeq* of version 0.9.1 [14]. In our analysis, we excluded 43 subjects including 12 male subjects and 31 subjects with >1% missing values. In addition, we removed the effects due to different age groups and batches using a median- matching and variance-matching strategy [10,15,16]. For example, the batch effect can be removed in the following way:

$$g_{ijk}^{*}=M_{i}+\left(g_{ijk}-M_{ij}\right)\frac{{\hat{\sigma }}_{g_{i}}}{{\hat{\sigma }}_{g_{ij}}},$$

where $g_{ijk}$ refers to the expression value for gene *i* from sample *k* in batch *j* ($j=1,2,...,J;k=1,2,...,n_{j}$), $M_{ij}$ represents the median of $g_{ij}=\left(g_{ij1},...,g_{ijn_{j}}\right)$, $M_{i}$ refers to the median of $g_{i}=\left(g_{i1},...,g_{iJ}\right)$, ${\hat{\sigma }}_{g_{i}}$ and ${\hat{\sigma }}_{g_{ij}}$ stand for the standard deviations of $g_{i}$ and $g_{ij}$, respectively.

The remaining 959 breast cancer samples were further classified into five subtypes according to two molecular signatures, namely *PAM50* [17] and *SCMOD2* [18]. The two classifications were implemented separately using R package *genefu* [19] and we obtained 530 subjects with concordant classification by two classifiers. The resulting set contains 221 subjects in the luminal A group, 119 in the luminal B group, 74 in the her2-enriched group, 105 in the basal-like group, and 11 in the normal-like group. For illustration purposes, we conducted two pairwise comparisons (1) Luminal A vs basal-like and (2) Luminal B vs basal-like.

**Figure 6:**
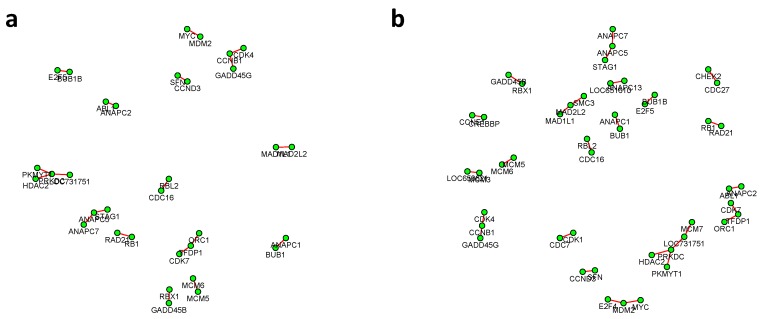

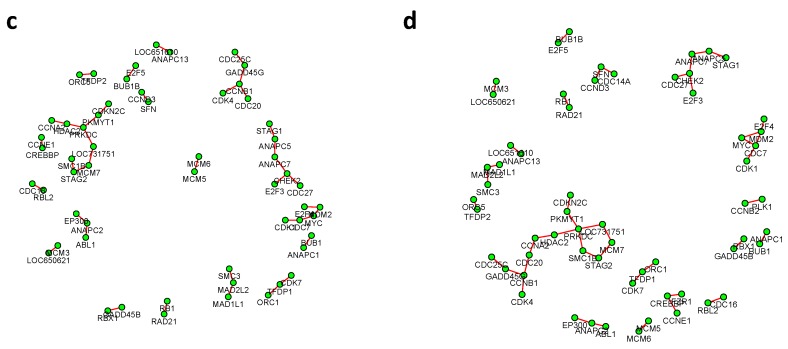
The inferred differential networks between the LumA and Basal-like subtypes under different desired FDR levels: (**a**) 0.05; (**b**) 0.10; (**c**) 0.15; (**d**) 0.20, with all isolated genes being removed. Each connection in the network represents an identified differential edge.

**Figure 7:**
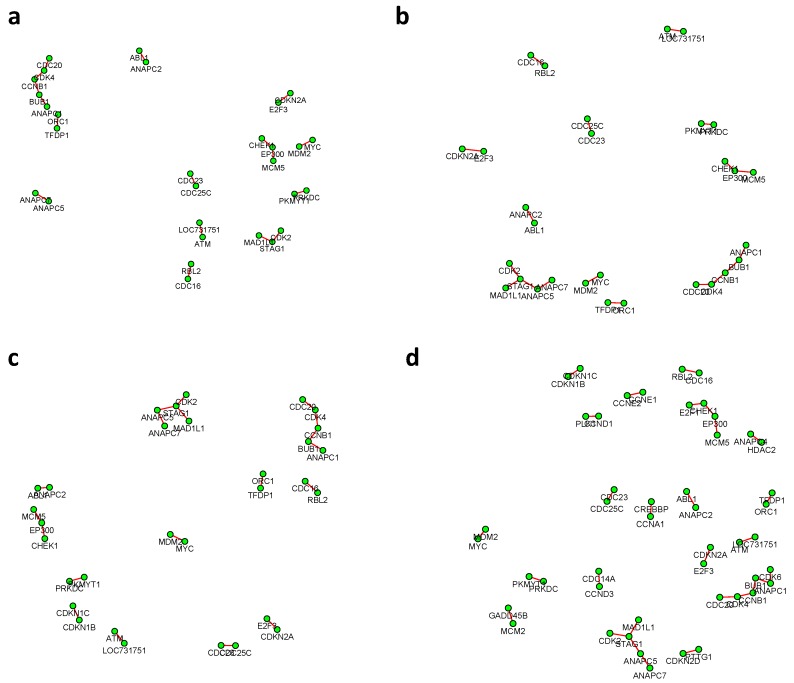
The inferred differential networks between the LumB and Basal-like subtypes under different desired FDR levels: (**a**) 0.05; (**b**) 0.10; (**c**) 0.15; (**d**) 0.20, with all isolated genes being removed. Each connection in the network represents an identified differential edge.

**Figure 8:**
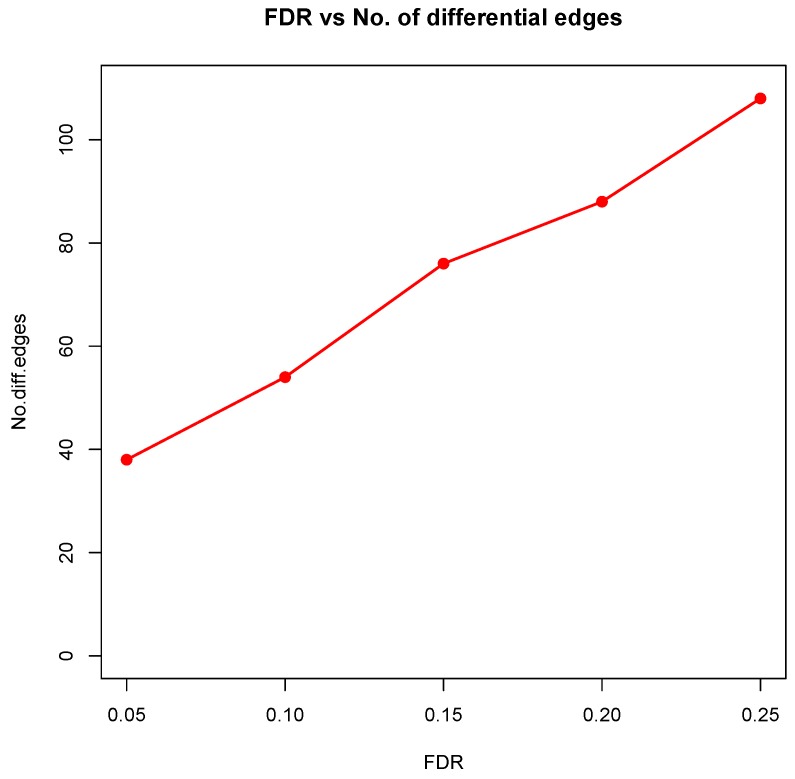
Desired FDR level against the number of differential edges.

To balance the bias and variance, we choose the same truncation parameter in Winsorized imputation as in our simulation study

$$\delta_{n}^{\left(k\right)}=\frac{1}{4n_{k}^{1/4}\sqrt{\pi \log n_{k}}},$$

where k∈{1,2}, $n_{1}=221$, $n_{2}=105$. The proposed test based on the Winsorized estimator was then conducted for each gene pair with different FDR cutoffs. Figure 6 and Figure 7 summarized all the identified differential edges under FDR levels α=0.05,0.10,0.15,0.20, with all isolated genes being removed. Our results suggested a list of important genes that play different roles in different breast cancer subtypes. For instance, in Figure 6, genes *CCNB1* and *PRKDC* contribute to several differential edges. According to recent studies, gene *CCNB1* is a prognostic biomarker for certain subtypes of breast cancer and it is closely associated with hormone therapy resistance [20]. It has also been reported in the literature that the *PRKDC* regulates chemosensitivity and is a potential prognostic and predictive marker of response to adjuvant chemotherapy in breast cancer patients [21]. Our findings about several other genes including *CHEK2* and *CDC7* also confirmed some existing reports [22,23]. As we observed from the two examples, as the desired FDR level increases, the resulting differential network tends to be denser and denser (Figure 8 showed the correlation between FDR and the number of differential edges). In practice, users should consider the trade-off between the accuracy (FDR) and number of new hypotheses (number of differential edges) and choose an appropriate FDR [24].

## 5. Discussion

Detecting the differential substructure on multiple graphical models is a fundamental and challenging problem in statistics. Liu (2017) studied the problem under the Gaussian framework and introduced an elegant hierarchical test based on the estimation of single GGM. Unlike most existing methods, Liu’s approach asymptotically controlled the false discovery rate at a nominal level, which guarantees the quality of the estimated differential network. In this work, we further extended Liu’s test to a more flexible semiparametric framework, namely the nonparanormal graphical models. Our test is built upon a Winsorized estimator of the unknown transformation functions and it enjoys similar asymptotic properties as its oracle counterpart does.

Although the new test holds great promise in many applications such as genetic network modeling, it has some practical limitations. First, as we see from the theoretical derivation, the good performance of the test relied on the sparsity assumption on the differential network. Although the sparsity assumption is reasonable in many cases, it still could be violated in some applications. For instance, some genetic pathways may exhibit a global change of gene–gene regulations between different phenotypes. When the differential network is dense or locally dense, the method may fail to control the FDR. To solve the problem, a new test needs to be defined to evaluate the level of the sparseness of the change between two conditions. However, there is still a gap on the literature of this topic.

Second, one key assumption in NPNGMs is that the transformed variables follow a joint Gaussian distribution. This assumption also needs to be checked in real-world applications. Under low dimensions, one can employ some popular normality tests, including the Anderson–Darling test and Shapiro–Wilk test, on the imputed data or other normal scores. However, most of these tests fail to detect non-normality for high-dimension data. The normality test under high dimension is still an open and challenging problem and we left it for future research.

It is also noteworthy to mention that the new test relied on an accurate estimator for the coefficients β. Motivated by [6], we chose two popular estimators including lasso estimator and Dantzig selector based on the adjusted Spearman’s rank, which satisfies Condition (7). In fact, some other estimators also satisfy the conditions, for instance, the rank-based adaptive lasso [6,25] and square-root lasso estimator [6,26]. These estimators can also be incorporated into our testing framework.

## 6. Conclusions

We have introduced a novel statistical test to detect the structural difference between the two nonparanormal graphical models. The proposed test dropped the Gaussian assumption and can be potentially applied to many non-Gaussian data for differential network analysis. For instance, some digital gene expression data (e.g., RNA-seq data) do not follow Gaussian distribution even after log transformation or other variance-stabilizing transformations. In such cases, one can model the data with a nonparanormal graphical model and apply our test to find differential edges between two or multiple phenotypic conditions. The proposed test may also be used to detect the difference between normal and disease populations in the brain connectivity network.

## Figures and Tables

**Table 1:** Running time of the proposed test and direct estimator (in seconds).

*n/p*	40	60	90	120
25	0.88 (7.0)	1.59 (110)	3.79 (1936)	6.49 (23,066)
50	1.19 (7.7)	1.93 (127)	4.15 (1973)	6.83 (23,119)
100	1.87 (9.1)	2.61 (146)	5.00 (2016)	7.80 (23,153)
200	2.11 (11)	3.04 (165)	6.22 (2055)	9.61 (23,201)

## Abbreviations

The following abbreviations are used in this manuscript:

FDR	False discovery rate
NPNGM	Nonparanormal graphical model
GGM	Gaussian graphical model
TCGA	The Cancer Genome Atlas

## Appendix A. Proof of Equation (6)

Define the estimated residuals based on Winsorized estimator as:

$$\epsilon_{mj}^{*}=X_{mj}^{*}-{\bar{X}}_{j}^{*}-\left(X_{m,-j}^{*}-{\bar{X}}_{-j}^{*}\right){\hat{\beta }}_{j},$$

where ${\bar{X}}_{j}^{*}=1/n\sum_{m=1}^{n}X_{mj}^{*}$ and ${\bar{X}}_{-j}^{*}=1/n\sum_{m=1}^{n}X_{m,-j}^{*}$. The choice of ${\hat{\beta }}_{j}$ must satisfy the following two conditions:

$${∥{\hat{\beta }}_{j}-\beta_{j}∥}_{\ell_{1}}=O_{p}\left(a_{n}\right),$$

$$\min\left\{\lambda_{\max}^{1/2}\left(\Sigma \right){∥{\hat{\beta }}_{j}-\beta_{j}∥}_{\ell_{2}},\max_{1\leq j\leq p}\sqrt{{\left({\hat{\beta }}_{j}-\beta_{j}\right)}^{T}{\hat{\Sigma }}_{-j,-j}\left({\hat{\beta }}_{j}-\beta_{j}\right)}\right\}=O_{p}\left(b_{n}\right),$$

$$\mathrm{where}\;a_{n}^{\left(k\right)}=o\left(\sqrt{\log p/n_{k}}\right),\;\;\mathrm{and}\;\;b_{n}^{\left(k\right)}=o\left(n_{k}^{-1/4}\right).$$

It is noteworthy to mention that the conditions above are slightly different from the conditions in [4] due to the different convergence rates by oracle data and imputed data. The conditions above can be satisfied by the rank-based estimators introduced in [6], e.g., rank-based lasso estimator or rank-based Dantzig selector. By letting ${\tilde{\epsilon }}_{mj}=\epsilon_{mj}-{\bar{\epsilon }}_{i}$, we have:

(A1) $$\begin{array}{cc} \epsilon_{mi}^{*}\epsilon_{mj}^{*}={\tilde{\epsilon }}_{mi}{\tilde{\epsilon }}_{mj} & -{\tilde{\epsilon }}_{mi}\left\{\left(X_{m,-j}^{*}-{\bar{X}}_{-j}^{*}\right){\hat{\beta }}_{j}-\left(X_{m,-j}-{\bar{X}}_{-j}\right)\beta_{j}\right\} \end{array}$$

(A2) $$\begin{array}{cc} \; & -{\tilde{\epsilon }}_{mj}\left\{\left(X_{m,-i}^{*}-{\bar{X}}_{-i}^{*}\right){\hat{\beta }}_{i}-\left(X_{m,-i}-{\bar{X}}_{-i}\right)\beta_{i}\right\} \end{array}$$

(A3) $$\begin{array}{cc} \; & +\left\{{\hat{\beta }}_{i}^{T}{\left(X_{m,-i}^{*}-{\bar{X}}_{-i}^{*}\right)}^{T}\left(X_{m,-j}^{*}-{\bar{X}}_{-j}^{*}\right){\hat{\beta }}_{j}-\beta_{i}^{T}{\left(X_{m,-i}-{\bar{X}}_{-i}\right)}^{T}\left(X_{m,-j}-{\bar{X}}_{-j}\right)\beta_{j}\right\}. \end{array}$$

First, for term (A3), we have:

$$\begin{array}{cc} \; & |\frac{1}{n}\sum_{m=1}^{n}\left\{{\hat{\beta }}_{i}^{T}{\left(X_{m,-i}^{*}-{\bar{X}}_{-i}^{*}\right)}^{T}\left(X_{m,-j}^{*}-{\bar{X}}_{-j}^{*}\right){\hat{\beta }}_{j}-\beta_{i}^{T}{\left(X_{m,-i}-{\bar{X}}_{-i}\right)}^{T}\left(X_{m,-j}-{\bar{X}}_{-j}\right)\beta_{j}\right\}|\\ = & |{\hat{\beta }}_{i}^{T}\left({\tilde{\Sigma }}_{-i,-j}-{\hat{\Sigma }}_{-i,-j}\right){\hat{\beta }}_{j}+{\left({\hat{\beta }}_{i}-\beta_{i}\right)}^{T}\left({\hat{\Sigma }}_{-i,-j}-\Sigma_{-i,-j}\right)\left({\hat{\beta }}_{j}-\beta_{j}\right)+{\left({\hat{\beta }}_{i}-\beta_{i}\right)}^{T}\Sigma_{-i,-j}\left({\hat{\beta }}_{j}-\beta_{j}\right)|\\ \leq & \max_{i,j}|{\hat{\beta }}_{i}^{T}\left({\tilde{\Sigma }}_{-i,-j}-{\hat{\Sigma }}_{-i,-j}\right){\hat{\beta }}_{j}|+\max_{i,j}|{\left({\hat{\beta }}_{i}-\beta_{i}\right)}^{T}\left({\hat{\Sigma }}_{-i,-j}-\Sigma_{-i,-j}\right)\left({\hat{\beta }}_{j}-\beta_{j}\right)|+\max_{i,j}\left|{\left({\hat{\beta }}_{i}-\beta_{i}\right)}^{T}\Sigma_{-i,-j}\left({\hat{\beta }}_{j}-\beta_{j}\right)\right|, \end{array}$$

where the last term can be bounded as follows:

$$\max_{i,j}|{\left({\hat{\beta }}_{i}-\beta_{i}\right)}^{T}\Sigma_{-i,-j}\left({\hat{\beta }}_{j}-\beta_{j}\right)|=O_{p}(\lambda_{\max}\left(\Sigma \right)\max_{1\leq i\leq p}∥{\hat{\beta }}_{i}-\beta_{i}{∥}_{\ell_{2}}^{2})=O_{p}\left(b_{n}^{2}\right).$$

It is not hard to show that:

$${∥\hat{\Sigma }-\Sigma ∥}_{\infty }=O_{p}\left(\sqrt{\frac{\log p}{n}}\right),$$

therefore, the second term can also be bounded

$$\max_{i,j}\left|{\left({\hat{\beta }}_{i}-\beta_{i}\right)}^{T}\left({\hat{\Sigma }}_{-i,-j}-\Sigma_{-i,-j}\right)\left({\hat{\beta }}_{j}-\beta_{j}\right)\right|=O_{p}\left(a_{n}^{2}\sqrt{\frac{\log p}{n}}\right).$$

Under some mild regularity conditions (stated in [6]), we have

$${∥\tilde{\Sigma }-\hat{\Sigma }∥}_{\infty }=O_{p}\left(\sqrt{\frac{\log p\log^{2}n}{n^{1/2}}}\right),$$

thus under the condition that $\max_{i,j}\left|\beta_{i,j}\right|\leq C_{1}$ and $\lambda_{\min}\left(\Sigma \right)=o\left({\left(\log p/n\right)}^{\frac{3}{4}}\right)$, the first term can be bounded as follows:

$$\begin{array}{cc} \; & \max_{i,j}\left|{\hat{\beta }}_{i}^{T}\left({\tilde{\Sigma }}_{-i,-j}-{\hat{\Sigma }}_{-i,-j}\right){\hat{\beta }}_{j}\right|\\ \leq & \max_{i,j}|\beta_{i}^{T}\left({\tilde{\Sigma }}_{-i,-j}-{\hat{\Sigma }}_{-i,-j}\right)\beta_{j}|+\max_{i,j}\left|{\left({\hat{\beta }}_{i}-\beta_{i}\right)}^{T}\left({\tilde{\Sigma }}_{-i,-j}-{\hat{\Sigma }}_{-i,-j}\right)\left({\hat{\beta }}_{j}-\beta_{j}\right)\right|\\ = & O_{p}\left(\sqrt{\frac{\log^{2}p\log^{2}n}{n^{3/2}}}+a_{n}^{2}\sqrt{\frac{\log p\log^{2}n}{n^{1/2}}}\right). \end{array}$$

Combining the three terms above, we have

$$\begin{array}{cc} \; & |\frac{1}{n}\sum_{m=1}^{n}\left\{{\hat{\beta }}_{i}^{T}{\left(X_{m,-i}^{*}-{\bar{X}}_{-i}^{*}\right)}^{T}\left(X_{m,-j}^{*}-{\bar{X}}_{-j}^{*}\right){\hat{\beta }}_{j}-\beta_{i}^{T}{\left(X_{m,-i}-{\bar{X}}_{-i}\right)}^{T}\left(X_{m,-j}-{\bar{X}}_{-j}\right)\beta_{j}\right\}|\\ = & O_{p}\left(\sqrt{\frac{\log^{2}p\log^{2}n}{n^{3/2}}}+a_{n}^{2}\sqrt{\frac{\log p\log^{2}n}{n^{1/2}}}+b_{n}^{2}\right). \end{array}$$

Next, we bound term (A1), which can be rewritten as:

$${\tilde{\epsilon }}_{mi}\left(X_{m,-j}-{\bar{X}}_{-j}\right)\left({\hat{\beta }}_{j}-\beta_{j}\right)+{\tilde{\epsilon }}_{mi}\left\{\left(X_{m,-j}-{\bar{X}}_{-j}\right)-\left(X_{m,-j}^{*}-{\bar{X}}_{-j}^{*}\right)\right\}{\hat{\beta }}_{j},$$

where the first term can be further decomposed into two parts,

$${\tilde{\epsilon }}_{mi}\left(X_{m,-j}-{\bar{X}}_{-j}\right)\left({\hat{\beta }}_{j}-\beta_{j}\right)={\tilde{\epsilon }}_{mi}\left(X_{mi}-{\bar{X}}_{i}\right)\left({\hat{\beta }}_{i,j}-\beta_{i,j}\right)I\left\{i\neq j\right\}+\sum_{l\neq i,j}{\tilde{\epsilon }}_{mi}\left(X_{ml}-{\bar{X}}_{l}\right)\left({\hat{\beta }}_{l,j}-\beta_{l,j}\right).$$

To bound $\underset{l\neq i,j}{\sum }{\tilde{\epsilon }}_{mi}\left(X_{ml}-{\bar{X}}_{l}\right)\left({\hat{\beta }}_{l,j}-\beta_{l,j}\right)$, we use the independence between $\epsilon_{mi}$ and $X_{m,-i}$. It is easy to show that

$$\max_{l\neq i}\left|\frac{1}{n}\sum_{m=1}^{n}{\tilde{\epsilon }}_{mi}\left(X_{ml}-{\bar{X}}_{l}\right)\right|=O_{p}\left(\sqrt{\frac{\log p}{n}}\right),$$

which indicates that

$$\max_{i,j}\left|\frac{1}{n}\sum_{m=1}^{n}\left(\sum_{l\neq i,j}{\tilde{\epsilon }}_{mi}\left(X_{ml}-{\bar{X}}_{l}\right)\left({\hat{\beta }}_{l,j}-\beta_{l,j}\right)\right)\right|=O_{p}\left(a_{n}\sqrt{\frac{\log p}{n}}\right).$$

By the independence between $\epsilon_{mi}$ and $X_{m}^{*}-X_{m}$, it is not hard to show

$$\begin{array}{cc} \; & |\frac{1}{n}\sum_{m=1}^{n}{\tilde{\epsilon }}_{mi}\left\{\left(X_{m,-j}-{\bar{X}}_{-j}\right)-\left(X_{m,-j}^{*}-{\bar{X}}_{-j}^{*}\right)\right\}{\hat{\beta }}_{j}|\\ \leq & |\frac{1}{n}\sum_{m=1}^{n}{\tilde{\epsilon }}_{mi}\left\{\left(X_{m,-j}-{\bar{X}}_{-j}\right)-\left(X_{m,-j}^{*}-{\bar{X}}_{-j}^{*}\right)\right\}\beta_{j}\left|+\right|\frac{1}{n}\sum_{m=1}^{n}{\tilde{\epsilon }}_{mi}\left\{\left(X_{m,-j}-{\bar{X}}_{-j}\right)-\left(X_{m,-j}^{*}-{\bar{X}}_{-j}^{*}\right)\right\}\left(\beta_{j}-{\hat{\beta }}_{j}\right)|\\ = & O_{p}\left(\frac{\log p}{n}+a_{n}\sqrt{\frac{\log p}{n}}\right). \end{array}$$

Combing term (A1) and term (A2), we have

$$\begin{array}{cc} \frac{1}{n}\sum_{m=1}^{n}{\tilde{\epsilon }}_{mi}\{\left(X_{m,-j}^{*}-{\bar{X}}_{-j}^{*}\right){\hat{\beta }}_{j}-\left(X_{m,-j}-{\bar{X}}_{-j}\right)\}\beta_{j} & =\frac{1}{n}\sum_{m=1}^{n}{\tilde{\epsilon }}_{mi}\left(X_{mi}-{\bar{X}}_{i}\right)\left({\hat{\beta }}_{i,j}-\beta_{i,j}\right)I\left\{i\neq j\right\}\\ \; & +O_{p}\left(\frac{\log p}{n}+a_{n}\sqrt{\frac{\log p}{n}}\right). \end{array}$$

Summarizing all the results above, by Equations (22) and (23) of Liu (2013), we have

$$\begin{array}{cc} \frac{1}{n}\sum_{m=1}^{n}\epsilon_{mi}^{*}\epsilon_{mj}^{*}=\frac{1}{n}\sum_{m=1}^{n}{\tilde{\epsilon }}_{mi}{\tilde{\epsilon }}_{mj} & -\frac{1}{n}\sum_{m=1}^{n}{\tilde{\epsilon }}_{mi}\left(X_{mi}-{\bar{X}}_{i}\right)\left({\hat{\beta }}_{i,j}-\beta_{i,j}\right)I\left\{i\neq j\right\}\\ \; & -\frac{1}{n}\sum_{m=1}^{n}{\tilde{\epsilon }}_{mj}\left(X_{mj}-{\bar{X}}_{j}\right)\left({\hat{\beta }}_{j,i}-\beta_{j,i}\right)I\left\{i\neq j\right\}\\ \; & +O_{p}\left(\sqrt{\frac{\log^{2}p\log^{2}n}{n^{3/2}}}+a_{n}^{2}\sqrt{\frac{\log p\log^{2}n}{n^{1/2}}}+a_{n}\sqrt{\frac{\log p}{n}}+b_{n}^{2}\right). \end{array}$$

As $\frac{1}{n}\overset{n}{\underset{m=1}{\sum }}{\tilde{\epsilon }}_{mi}\left(X_{mi}-{\bar{X}}_{i}\right)=\frac{1}{n}\overset{n}{\underset{m=1}{\sum }}{\tilde{\epsilon }}_{mi}^{2}+\frac{1}{n}{\tilde{\epsilon }}_{mi}\left(X_{m,-i}-{\bar{X}}_{-i}\right)\beta_{i}$, and $\mathrm{Var}\left(X_{m,-i}\beta_{i}\right)=\left(\sigma_{ii}\omega_{ii}-1\right)/\omega_{ii}\leq C$, we have

$$\max_{1\leq i\leq p}\left|\frac{1}{n}\sum_{m=1}^{n}{\tilde{\epsilon }}_{mi}\left(X_{m,-i}-{\bar{X}}_{-i}\right)\beta \right|=O_{p}\left(\frac{\log p}{n}\right),$$

therefore

$$\begin{array}{cc} \frac{1}{n}\sum_{m=1}^{n}{\tilde{\epsilon }}_{mi}\left(X_{mi}-{\bar{X}}_{i}\right) & =\frac{1}{n}\sum_{m=1}^{n}{\tilde{\epsilon }}_{mi}^{2}+O_{p}\left(\log p/n\right)\\ \; & =\frac{1}{n}\sum_{m=1}^{n}\epsilon_{mi}^{*2}+O_{p}\left(\sqrt{\frac{\log^{2}p\log^{2}n}{n^{3/2}}}+a_{n}^{2}\sqrt{\frac{\log p\log^{2}n}{n^{1/2}}}+a_{n}\sqrt{\frac{\log p}{n}}+b_{n}^{2}\right), \end{array}$$

$$\begin{array}{cc} \frac{1}{n}\sum_{m=1}^{n}\epsilon_{mi}^{*}\epsilon_{mj}^{*} & =\frac{1}{n}\sum_{m=1}^{n}{\tilde{\epsilon }}_{mi}{\tilde{\epsilon }}_{mj}-\frac{1}{n}\sum_{m=1}^{n}\epsilon_{mi}^{*2}\left({\hat{\beta }}_{i,j}-\beta_{i,j}\right)I\left\{i\neq j\right\}-\frac{1}{n}\sum_{m=1}^{n}\epsilon_{mj}^{*2}\left({\hat{\beta }}_{j,i}-\beta_{j,i}\right)I\left\{i\neq j\right\}\\ \; & +O_{p}\left(\sqrt{\frac{\log^{2}p\log^{2}n}{n^{3/2}}}+a_{n}^{2}\sqrt{\frac{\log p\log^{2}n}{n^{1/2}}}+a_{n}\sqrt{\frac{\log p}{n}}+b_{n}^{2}\right). \end{array}$$

In addition

$$\frac{1}{n}\sum_{m=1}^{n}\epsilon_{mi}^{*2}=\frac{1}{n}\sum_{m=1}^{n}{\tilde{\epsilon }}_{mi}^{2}+O_{p}\left(\sqrt{\frac{\log^{2}p\log^{2}n}{n^{3/2}}}+a_{n}^{2}\sqrt{\frac{\log p\log^{2}n}{n^{1/2}}}+a_{n}\sqrt{\frac{\log p}{n}}+b_{n}^{2}\right).$$

Equation (6) follows immediately by central limit theorem.
